# Exact solutions for the 2d-strip packing problem using the positions-and-covering methodology

**DOI:** 10.1371/journal.pone.0245267

**Published:** 2021-01-14

**Authors:** Nestor M. Cid-Garcia, Yasmin A. Rios-Solis

**Affiliations:** 1 Laboratorio Nacional de Geointeligencia, CONACYT-Centro de Investigación en Ciencias de Información Geoespacial, Aguascalientes, Aguascalientes, Mexico; 2 Escuela de Ingeniería y Ciencias, Tecnológico de Monterrey, Monterrey, Nuevo León, Mexico; Torrens University Australia, AUSTRALIA

## Abstract

We use the *Positions and Covering* methodology to obtain exact solutions for the two-dimensional, non-guillotine restricted, strip packing problem. In this classical NP-hard problem, a given set of rectangular items has to be packed into a strip of fixed weight and infinite height. The objective consists in determining the minimum height of the strip. The *Positions and Covering* methodology is based on a two-stage procedure. First, it is generated, in a pseudo-polynomial way, a set of valid positions in which an item can be packed into the strip. Then, by using a set-covering formulation, the best configuration of items into the strip is selected. Based on the literature benchmark, experimental results validate the quality of the solutions and method’s effectiveness for small and medium-size instances. To the best of our knowledge, this is the first approach that generates optimal solutions for some literature instances for which the optimal solution was unknown before this study.

## Introduction

The *Two-Dimensional Strip Packing Problem* (2SP) is composed of a given set of *n* rectangular items, each one with specific width *w*_*i*_ and height *h*_*i*_, for *i* = 1, …, *n*, and a strip of width *W* and infinite height. The aim is to place all the items into the strip orthogonally; without overlapping, minimizing the overall strip’s height [1, 2]. We assume that all input data *w*_*i*_, *h*_*i*_, and *W* are positive integers and that *w*_*i*_ ≤ *W* for all items *i* = 1, …, *n*. We consider the case when the items have a fixed orientation, and the guillotine cut constraint is unnecessary.

**Fig 1:**
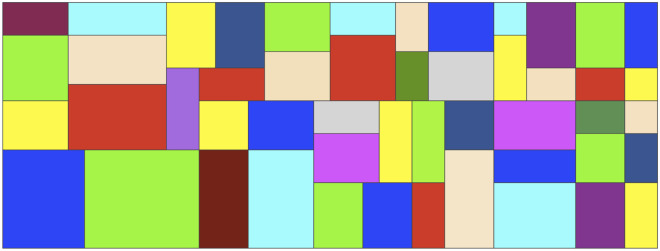
Example for 2SP. The optimal configuration for an instance proposed by [6] with 50 items, a strip of width *W* = 40, and an optimal height *H* = 15.

The 2SP is NP-hard in the strong sense since it can be reduced to the one-dimensional bin-packing problem [2–4], and according to the typology proposed by [5], the 2SP belongs to the class of cutting and packing problems: *two-dimensional, open dimension problem (2D-ODP)*. Fig 1 shows the optimal configuration for an instance proposed by [6] with 50 items and a strip of width *W* = 40. The optimal height is *H* = 15. In this case, there is no wasting in the strip; that is, we have a *perfect packing*.

Many real-world applications of this problem can be found in the paper, textile, glass, steel, and wood industries, where rectangular items are cut from larger rectangular sheets of material that can be considered with infinite height [7, 8]. The 2SP also appears in scheduling problems where the tasks require a contiguous subset of identical resources [9].

In this study, we propose an adaptation of the *Positions and Covering* (P&C) methodology, used by [10] to obtain optimal solutions for the two-dimensional bin packing problem. Considering the similarity of the 2SP with other packing problems, some potential applications for the P&C methodology can be found in agricultural fields to delineate rectangular and homogeneous management zones [11–13], in scheduling problems applied to environmental, automotive, ferry, and manufacturing industries to ensure maximum use of materials [14–16], in healthcare applied to operating rooms schedules [17–19], in cloud computing where a set of jobs must be processed on virtual machines of physical servers with the aim of improves the energy efficiency [20–22], and in optimal deliveries in e-commerce where the objective is optimizing the total number of containers to integrate into several delivery trips [23].

The P&C methodology adapted to the 2SP is as follows. Given an instance of the 2SP, the first step of the P&C is to compute the strip’s height with the assumption that a perfect packing exists. Then we generated a set of valid positions to determine all possible places to locate it on the strip for each item. This pre-processing is the key-point of the P&C methodology. Finally, using the *H* computed, the P&C solves a set-covering model for the decision version of the 2SP (D-2SP(*H*)): is there a non-overlapping packing of the *n* items into the strip with *H* height? If there is a feasible solution, then *H* is the optimal value for the 2SP. Otherwise, P&C iterates again, increasing the height *H* of the strip by one. The P&C is an exact methodology that obtains optimal solutions for the 2SP, that is, every time our methodology is executed, the same solution and time are going to be obtained.

The main differences of our methodology with respect to the mentioned exact approaches is that the P&C groups the items with identical size and computes their demand. Indeed, all the other approaches consider similar items as individual. This grouping makes a better covering model that can be solved faster. Moreover, this new set-covering formulation is strengthened with two families of valid inequalities. This manner, the P&C methodology is power enough to obtain optimal solutions for the 2SP problem without any more complex methodology as column generation.

Because of the combinatorial complexity of the 2SP, the attempts to solve it are roughly divided into exact and approximation methods. Some reviews of the 2SP are presented in [8, 24, 25], and some surveys for packing and cutting problems are showed in [26–29].

In terms of other exact methods, there have been recent combinatorial branch-and-bound (B&B) algorithms that build solutions by packing items one at a time in the strip like the ones of [1, 2, 30–33]. In [1], the authors propose a B&B algorithm to solve the 2D rectangular packing problems, a particular case of the 2SP. This B&B algorithm is enhanced with a dynamic programming mechanism for determining if gaps can be filled.

A new relaxation to produce good lower bounds and obtain practical heuristic algorithms is introduced in [2]. These bounds were also used in a B&B algorithm. The authors of [33] propose two algorithms for the 2SP with and without 90-degrees rotations. They are based on a branching operation that uses the staircase placement. Two exact algorithms and an approximate algorithm have been proposed by [34] to solve a variant of the strip cutting problem. These algorithms are based on B&B and dynamic programming procedures.

Concerning the heuristics and metaheuristics methods to solve the 2SP, in [3], the authors introduce the bottom-left (BL) heuristic. Some approaches with variants or implementations of the BL strategy to solve packing problems can be found in [6, 35–39]. Other works that implement metaheuristics methods as tabu-search, simulated annealing, and genetic algorithms are presented in [40–43].

In [44], the authors propose two metaheuristics that involve the application of the simulated annealing with a heuristic construction algorithm. In many of these studies, the authors use a version of the BL heuristic to arrange the items. In [45] the authors show some models strengthen with well-known valid inequalities and based on the work of [43]. Some improvements for the best-fist heuristic, where it is presented a simple local random local search, are showed in the work of [46]. The use of data mining techniques also has been implemented to assess the quality of heuristics solutions [47]. Other approaches that consider hierarchical or multi-stage methodologies can be found in the works of [48–50]. A survey of heuristics for the two-dimensional rectangular strip packing problem is presented in [51], and upper bounds for heuristic approaches in [52].

The main strength of the P&C methodology for the 2SP is that it obtains optimal solutions for many instances of the classical benchmarks that none of the previous (and more elaborated) methods could find. For example, the P&C solved to optimality several instances proposed by [53] for which the optimal solutions were not known before of this study. To the best of our knowledge, the P&C methodology is one of the best methods to find optimal solutions for the 2SP for small and medium-size instances.

The rest of this article is organized as follows. In Section *Materials and methods*, we describe the P&C methodology for the 2SP. In Section *Results*, we present the experimental results for the P&C methodology by using a set of small and medium-size instances that have been broadly used in the literature to test other algorithms. Finally, in Section *Conclusions*, we make some concluding remarks.

## Materials and methods

In this section, we present the materials and methods used to solve the 2SP. First, we show the adaptation of the P&C proposed by [10] to solve the 2d-bin packing problem. Then, we describe each part of the methodology for the 2SP.

### The P&C methodology for the 2SP

**Fig 2:**
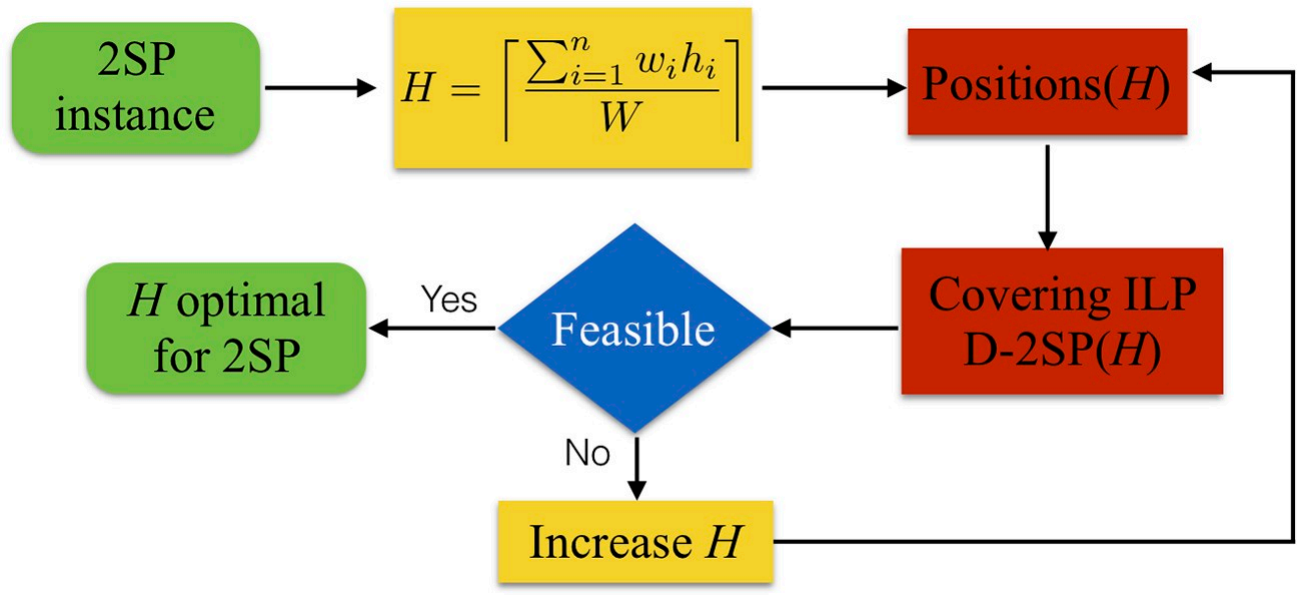
Adaptation of the P&C methodology. The two-step methodology to solve the 2SP.

The adaptation of the P&C methodology for the 2SP is schematized in Fig 2. Given an instance of the 2SP, the first step is to compute the height *H* of the strip, assuming that a perfect packing exists, that is, the total area of the items divided by the width of the strip, as shown in Eq (1). Notice that if a better lower bound of the 2SP instance is known, then this first value of the height could be updated, and some iterations of the P&C may be avoided.

$$\begin{array}{c} H=\lceil \frac{\sum_{i=1}^{n}w_{i}h_{i}}{W}\rceil \end{array}$$ (1)

Considering this *H*-value, the P&C generates the set of valid positions that locate the items inside of the strip. This step is the key-point of our methodology, and it will be later explained in detail. Then, an integer linear programming set-covering formulation (ILP) for the decision version of the 2SP, named as D-2SP(*H*), is solved to optimality. If the covering model is feasible, then *H* is the optimal height of the strip. Else, the *H*-value is increased by one or in a dichotomous way, the new set of valid positions for the items is generated, and the covering model is solved again. These last three steps represent the main difference with the P&C for the 2D-BPP (see [10]). The procedure ends when P&C finds a feasible solution of the D-2SP(*H*), and the current height becomes the optimal one.

### Positions stage for the 2SP

The objective of the *Positions* stage is to generate the set of *valid positions* where an item can be placed into the strip, that is, from the infinite set of positions that an item can take in the bin, the P&C determines only a finite set that guarantees the optimality of the solution. The inputs of the Positions stage are i) the number *n* of items with their specifics width *w*_*i*_, height *h*_*i*_, and demand *d*_*i*_, ii) the width *W* of the strip, and iii) the current height *H* of the strip (first computed with Eq 1 or a known lower bound, or an updated value).

**Fig 3:**
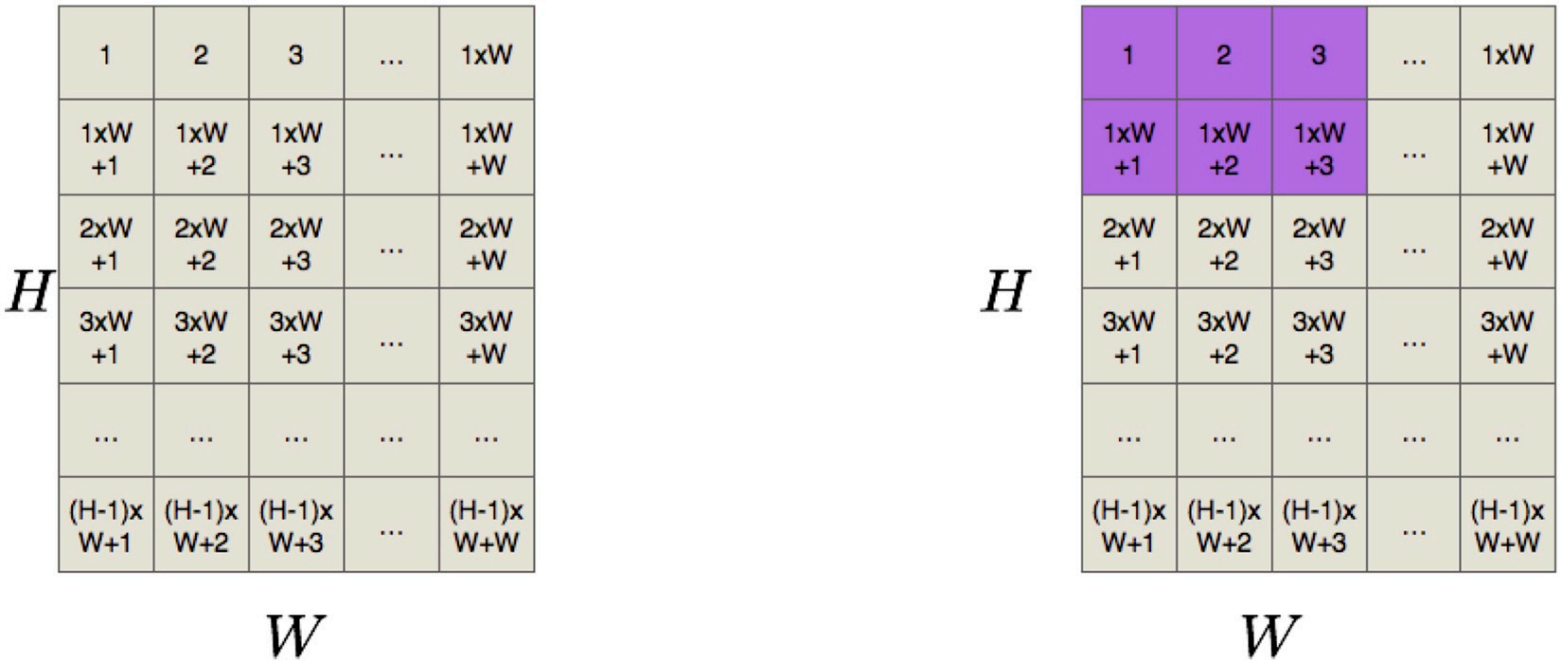
First step of the P&C methodology. a) Grid inside of the strip and b) Valid position for an item with dimensions 2 × 3 with its top-left corner at tile 1.

With the current height *H* of the strip, the first step in the *Positions* stage is to delineate a Cartesian grid inside the strip, that is, a regular tessellation of the 2-dimensional Euclidean space by congruent unit squares, where each square has a particular identification. We arbitrarily choose the enumeration that starts at the top left corner square and ends at the bottom right square (see Fig 3a). Thus, for each item, a valid position is created if its top left corner coincides with a tiling, and its width and height dimensions do not exceed the size of the strip. Each valid position is labeled to differentiate one from each other. Fig 3b shows the valid position for an item with dimensions 2 × 3 with its top-left corner at tile 1. Notice that the position which starts in the tile 1 × *W* would not be a valid position for this item. Let *J*^*H*^ be the set of valid positions for all the items for a fixed height *H* of the strip. To generate *J*^*H*^, we start populating its valid positions width-wise and then length-wise for each item. The size of *J*^*H*^ is pseudo-polynomial, but this pre-processing allows the P&C method to decompose the problem and to reach optimal solutions.

**Fig 4:**
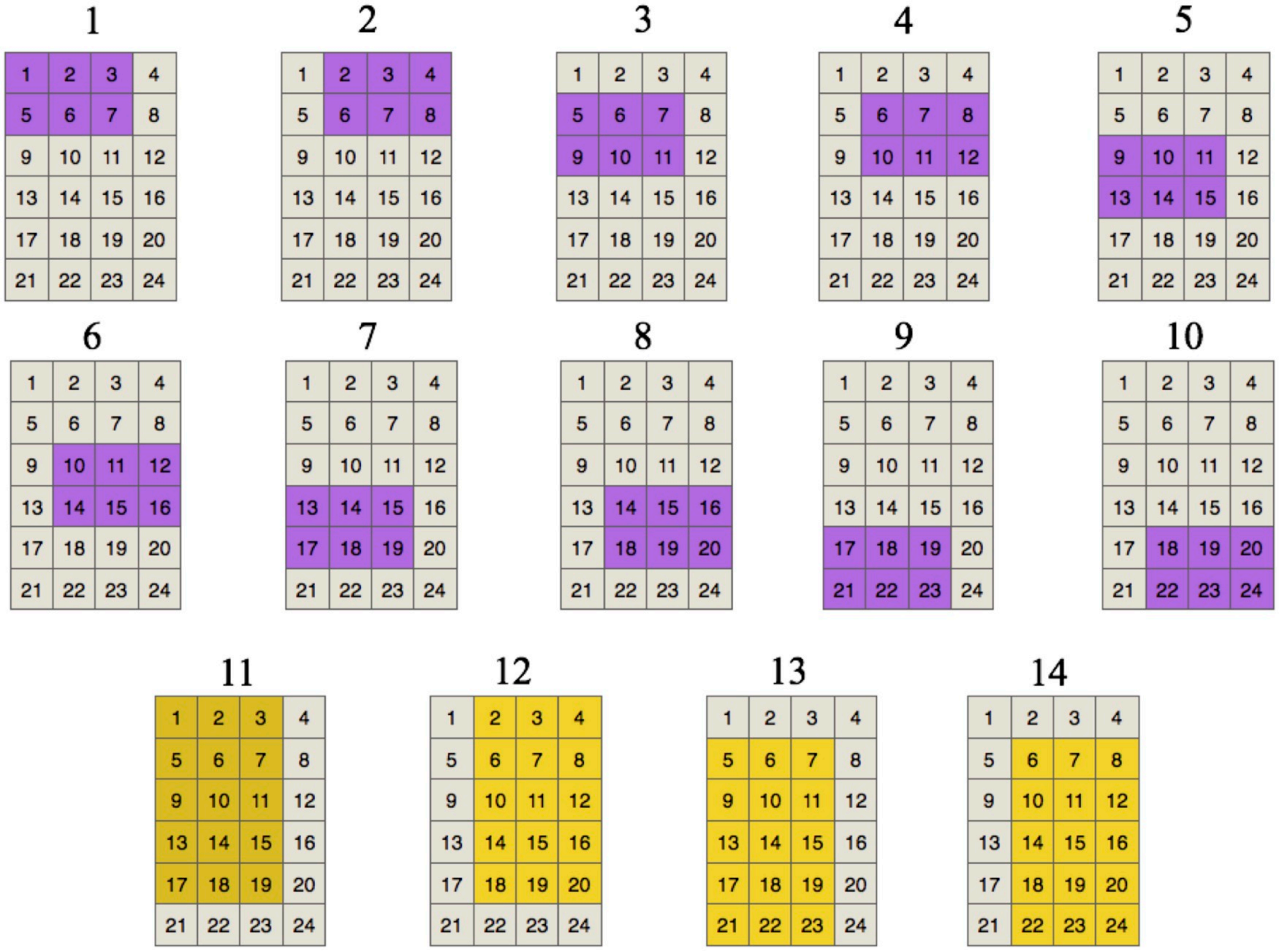
Generation of positions. Set *J*^*H*^ of valid positions for items of 2 × 3 and 5 × 3 in a strip with *H* = 6 and *W* = 4.

Fig 4 shows the set of valid positions for an item of 2 × 3 and an item of 5 × 3 in a strip with dimensions 6 × 4. In this case, *J*^*H*^ has 14 valid positions. The set from 1 to 10 corresponds for the first item while the set from 11 to 14 to the second item. Each valid position is unique; therefore, it has a specific label and an unrepeatable tiles set. For example, position 1 (corresponding to the 2 × 3 item) contains tiles 1, 2, 3, 5, 6, and 7.

The resulting set of valid positions may be view as a correspondence matrix *C*^*H*^ = {*c*_*jp*_}, where rows represent the set of valid positions *j* ∈ *J*^*H*^ and columns are the tiles in the strip. Matrix *C*^*H*^ is composed of 1’s and 0’s, where *c*_*jp*_ = 1 if valid position *j* covers tile *p*, *c*_*jp*_ = 0 otherwise. The correspondence matrix of Fig 4 (set *J*^*H*^ for an item of 2 × 3 and another one of 5 × 3 in a strip with *H* = 6 and *W* = 4) appears in Table 1. Each row in the correspondence matrix represents the respective valid position presented in Fig 4. For example, row 1 has ones in tiles 1, 2, 3, 5, 6, and 7, which correspond with position 1 of the figure.

**Table 1:** Correspondence matrix *C*^*H*^ for the set *J*^*H*^ of two, one of 2 × 3 and the other of 5 × 3, in a strip with *H* = 6 and *W* = 4, corresponding to Fig 4.

		Tiles on the strip (*p*)															
		1	2	3	4	5	6	7	8	9	10	11	12	13	14	…	24
Valid positions (*j*)	1	1	1	1	0	1	1	1	0	0	0	0	0	0	0	…	0
	2	0	1	1	1	0	1	1	1	0	0	0	0	0	0	…	0
	3	0	0	0	0	1	1	1	0	1	1	1	0	0	0	…	0
	4	0	0	0	0	0	1	1	1	0	1	1	1	0	0	…	0
	5	0	0	0	0	0	0	0	0	1	1	1	0	1	1	…	0
	6	0	0	0	0	0	0	0	0	0	1	1	1	0	1	…	0
	7	0	0	0	0	0	0	0	0	0	0	0	0	1	1	…	0
	8	0	0	0	0	0	0	0	0	0	0	0	0	0	1	…	0
	9	0	0	0	0	0	0	0	0	0	0	0	0	0	0	…	0
	10	0	0	0	0	0	0	0	0	0	0	0	0	0	0	…	1
	11	1	1	1	0	1	1	1	0	1	1	1	0	1	1	…	0
	12	0	1	1	1	0	1	1	1	0	1	1	1	0	1	…	0
	13	0	0	0	0	1	1	1	0	1	1	1	0	1	1	…	0
	14	0	0	0	0	0	1	1	1	0	1	1	1	0	1	…	1

Notice that the set of valid positions determined by the unitary grid is sufficient to reach the optimal solution of the 2SP since all input data are integer.

### Covering stage

In this stage, the P&C executes a set-covering formulation based on an integer linear programming model. This formulation solves the decision problem for the strip packing problem D-2SP(*H*), that is, is there a solution for the 2SP when the height is set to *H*? If the answer is positive, then the procedure ends (since we are minimizing the height), else the height of the strip is increased by one. A new iteration begins by populating the new set of valid positions for each item, and the decision model is solved again.

Formalizing, let *I* be the set of items, recall that *J*^*H*^ is the set of valid positions for a fixed height of the strip, *V*(*i*) be the subset of valid positions for each item *i* ∈ *I* where *V*(*i*)∈*J*, and *P* be the tiles set inside of the strip. Furthermore, we know other parameters such as the demand *d*_*i*_ of item *i* ∈ *I* and the maximum of times that this item can be packed inside the strip: *UB*_*i*_.

The decision variables for the ILP model are:

$$\begin{array}{c} x_{ij}=\{\begin{array}{cc} 1\; & \text{if}\;\text{item}\;i\;\text{is}\;\text{placed}\;\text{in}\;\text{valid}\;\text{position}\;j\in V(i)\text{,}\;\text{for}\;i\in I\text{,}\;\\ 0\; & \text{otherwise.}\; \end{array} \end{array}$$

This manner, the set-covering formulation to solve the decision problem D-2SP(*H*) is as follows.

$$\begin{array}{ccc} & \min\;z=0 & \end{array}$$ (2)

*s.t*.

$$\begin{array}{ccc} & \sum_{i\in I}\sum_{j\in V(i)}c_{jp}x_{ij}\leq 1 & p\in P, \end{array}$$ (3)

$$\begin{array}{ccc} & \sum_{j\in V(i)}x_{ij}\geq d_{i} & i\in I, \end{array}$$ (4)

$$\begin{array}{ccc} & \sum_{j\in V(i)}x_{ij}\leq UB_{i}\; & i\in I. \end{array}$$ (5)

$$\begin{array}{ccc} & \sum_{i\in I}\sum_{j\in V(i)}\sum_{p\in P}c_{jp}x_{ij}\leq WH & \; \end{array}$$ (6)

$$\begin{array}{ccc} & x_{ij}\in \left\{0,1\right\} & i\in I,j\in J \end{array}$$ (7)

where (2) establishes that any feasible solution for the 2SP is desirable. Constraints (3) avoid the overlapping assigning at most one item at each tile of the strip. Constraints (4) guarantee that all the items will be packed into the strip. Constraints (5) act as valid inequalities (valid by definition) since we bound the number of occurrences of each item in the strip. Constraint (6) determines that the capacity of the strip must not be exceeded; that is, it is impossible to pack more items than allowed. This constraint is also a valid inequality that strengthens the constraint space. Finally, in (7), the nature of the variables is declared.

Besides valid inequalities (5) and (6), other valid inequalities could enhance the ILP, but these proved in preliminary results to be the more efficient for the 2SP (and also for the 2BPP, see [10]).

## Results

To test the P&C methodology, we use two sets of instances: the original benchmark for the strip packing and the benchmark for other two-dimensional cutting problems. The description for each group of instances is given in the following sections. Then, we present the experimental results on these sets.

### Original instances for the strip packing

This instance set refers to the instances generated for the two-dimensional strip packing problem. This set contains:

- 2 perfect packing instances proposed by [6], known as jack01-jack02.
- 4 instances proposed by [53–55], known as dagli01-dagli04.
- 1 instance proposed by [56], known as kendall.
- 25 instances proposed by [34], known as hifi01-hifi25.
- 9 of 21 instances proposed by [36], known as hopper.

The optimum for instances dagli01, dagli03 and, dagli04 was not known until now, according to the Working Group on Cutting and Packing within the Association of the European Operational Research Societies (ESICUP) (https://www.euro-online.org/websites/esicup/data-sets/#1535972088188-55fb7640-4228).

For some instances like the hopper ones, we are no testing all of the instances of the benchmark since they are too large for the P&C methodology. Indeed, other instances benchmark exist in the literature but they are also too large for the P&C so they are not mentioned in this section.

### Instances for other two-dimensional cutting problems

This set of instances was originally introduced for other two-dimensional cutting problems and was transformed into strip packing instances considering the item size and the bin width. We found the following instances:

- 100 of 300 instances proposed by [57]. These instances are divided into 10 classes (6 and 4, respectively), where each class is composed of 5 groups of 10 instances. Each group has a different number of items to be packed into the bin with *n* = {20, 40, 60, 80, 100}. The corresponding best-known solution and lower bound for each instance are available at http://www.or.deis.unibo.it/research_pages/ORinstances/2BP.html. This set of instances is known as the *BW* instances.
- 3 instances proposed by [58], known as *cgcut*.
- 12 instances proposed by [59], known as *ngcut*. The *cgcut* and *ngcut* instances are test problems for 2D cutting problems, which were transformed to 2D bin packing instances according to [60]. These instances are available from the ORLIB library.
- 10 instances generated by [61], known as *beng*. They are vailable at PackLib2 ([62]), http://www.ibr.cs.tu-bs.de/alg/packlib/index.shtml.
- 6 instances proposed by [33], known as kenmochi.

As in the original instances for the strip packing problem, there are other set of instances that we do not include in this study since they are too large for the P&C.

### Computational results

To solve the set-covering formulation, we use the integer linear programming solver (B&B) of CPLEX 12.7 using its default options, except for the optimality parameter set to 0%. All the instances were executed on a computer equipped with a processor 8-Core Intel Xeon E5-2609 @1.70 GHz, and 64 GB of RAM. The time limit for the B&B execution was fixed to 8 hours (28800 seconds).

For all of the instances, we start the P&C methodology using the height of the strip *H* given by the best lower bound obtained in the literature. When this bound is not available, we compute the *H* parameter assuming that a perfect packing exists (Eq (1)). We report the execution time for the last iteration of the P&C.

We compare the P&C with several methodologies that were implemented and tested on computers with different characteristics. Thus, our comparison is informative more than comparative. The main purpose of this study is to obtain optimal solutions for instances of the 2SP. Furthermore, statistical tests are not required to determine the solution quality because P&C is an exact method without statistical components on its procedure.

#### Original instances for the strip packing

Tables 2–5 show the experimental results for the original instances of the 2SP. The structure of these tables is as follows: the first three columns describe the instance, that is, the name (“Inst”), the width size *W* of the strip, and the number *n* of items. The fourth column indicates the optimum (“Opt”) or the best lower bound known (“LB”) in the literature. Columns 5–7 present the values for the P&C methodology: the generation time in seconds of the correspondence matrix (“GT”), the execution time in seconds required by the B&B solver (“ET”), and the optimum value z*. In the last columns, we present the solutions obtained by other approaches in the literature.

**Table 2:** Results for jack and kendall instances.

Inst	Size		Opt	P&C			Jakobs	Liu and Teng	Mumford–Valenzuela	Bortfeldt
	*W*	*n*		GT	ET	z*				
jack01	40	25	15	0.59	22.40	**15**	17	16	16	16
jack02	40	50	15	0.71	14.70	**15**	17	16	16	**15**
kendall	80	13	140	65.70	1.59	**140**				
Number of optimal solutions						3/3	0/3	0/3	0/3	1/3

**Table 3:** Results for dagli instances.

Inst	Size		LB	P&C					Ratanapan and Dagli	Dagli and Poshyanonda	Bortfeldt
	*W*	*n*		GT	ET	z*	%	$\bar{LB}$			
dagli01	60	31	45	4.21	843.86	**46**	100		91.88	–	95.67
dagli02	60	21	40	1.87	4.41	**40**	100		92.50	–	97.56
dagli03	30	37	112	8.02	78.78	**112**	100		94.41	96.03	98.58
dagli04	20	37	161	10.43	17.89	209	100	208	–	97.15	97.62
Number of optimal solutions						3/4			0/4	0/4	0/4

A “-” mark means that the authors did not solve this particular instance.

**Table 4:** Results for hifi instances.

Inst	Size		LB	P&C			Hifi		*S*_*bb*_		*S*_*da*_		*S*_*bp*_	
	*W*	*n*		GT	ET	z*	*z*	ET	*z*	ET	*z*	ET	*z*	ET
hifi01	5	10	13	0.01	0.00	**13**	**13**	<0.10	**13**	0.00	**13**	0.00	**13**	0.00
hifi02	4	11	40	0.01	0.08	**40**	**40**	0.30	**40**	0.00	**40**	0.00	**40**	0.00
hifi03	6	15	14	0.01	0.06	**14**	**14**	0.30	**14**	7.10	**14**	403.83	19	0.00
hifi04	6	11	19	0.01	0.02	**20**	**20**	0.60	**20**	2.45	**20**	160.35	22	OM
hifi05	20	8	20	0.04	0.00	**20**	**20**	0.50	**20**	0.00	**20**	0.00	**20**	0.00
hifi06	30	7	38	0.58	0.01	**38**	**38**	4.90	**38**	0.00	**38**	0.00	**38**	0.00
hifi07	15	8	14	0.01	0.02	**14**	**14**	0.30	**14**	0.01	**14**	0.06	**14**	1.01
hifi08	15	12	17	0.02	0.05	**17**	**17**	0.50	**17**	1.57	**17**	10.45	**17**	0.00
hifi09	27	12	68	1.2	4.14	**68**	**68**	7.60	**68**	0.14	**68**	0.01	**68**	0.00
hifi10	50	8	80	3.72	20.84	**80**	**80**	0.30	**80**	0.18	**80**	0.01	**80**	0.00
hifi11	27	10	48	1.05	3.21	**48**	**48**	8.60	**48**	4.89	**48**	0.42	**48**	0.00
hifi12	81	18	34	3.36	0.03	**34**	**34**	2.80	38	3600	**34**	0.01	38	3600
hifi13	70	7	50	4.78	11.47	**50**	**50**	6.10	**50**	0.38	**50**	0.15	50	0.00
hifi14	100	10	60	12.31	77.63	**69**	**69**	4.10	**69**	15.57	**69**	196.65	69	0.00
hifi15	45	14	34	1.70	13.22	**34**	**34**	6.00	**34**	2688.13	**34**	445.94	**34**	0.00
hifi16	6	14	32	0.02	0.11	**33**	**33**	1.40	**33**	606.12	**33**	216.83	35	OM
hifi17	42	9	34	1.12	5.70	**34**	39	7.10	**34**	2.07	**34**	0.00	**34**	0.00
hifi18	70	10	89	17.38	2777.12	**100**	101	10.70	**100**	9.43	**100**	97.03	**100**	88.25
hifi19	5	12	25	0.01	0.08	**25**	26	0.30	26	0.01	26	16.36	27	OM
hifi20	15	10	19	0.05	0.29	**20**	21	1.80	**20**	1.73	**20**	1.32	**20**	0.00
hifi21	30	11	140	7.22	141.44	**140**	145	3.90	**140**	24.31	**140**	63.29	**140**	0.00
hifi22	90	22	34	6.70	35.57	**34**	**34**	11.80	42	3600	39	1597.91	43	3600
hifi23	15	12	34	0.08	0.21	**34**	35	1.50	**34**	37.97	**34**	24.07	39	0.00
hifi24	50	10	103	18.96	995.92	**109**	114	18.90	**109**	19.34	**109**	71.05	**109**	0.00
hifi25	25	15	35	0.82	14.04	**35**	36	11.50	36	3600	36	657.15	43	3600
Number of optimal solutions						25/25	17/25		21/25		22/25		15/25	

**Table 5:** Experimental results for hopper instances.

Class	Inst	Size		Opt	P&C			Iori	Best-fit	Burke			Bortfeldt		GRASP	
		*W*	*n*		GT	ET	z*			BF+TS	BF+SA	BF+GA	Avg	Best	Avg	Best
C1	01	20	16	20	0.21	0.00	**20**	**20**	21	**20**	**20**	**20**			**20**	**20**
	02	20	17	20	0.20	12.67	**20**	21	22	21	**20**	21			**20**	**20**
	03	20	16	20	0.19	2.42	**20**	**20**	24	**20**	**20**	**20**			**20**	**20**
Average percentage deviation from optimum							**0**	1.59	10.17	1.59	**0**	1.59	1.59	1.59	**0**	**0**
C2	04	40	25	15	0.73	26.05	**15**	**15**	16	16	16	16			**15**	**15**
	05	40	25	15	0.69	64.59	**15**	16	16	16	16	16			**15**	**15**
	06	40	25	15	0.66	16.39	**15**	**15**	16	16	16	16			**15**	**15**
Average percentage deviation from optimum							**0**	2.08	6.25	6.25	6.25	6.25	3.33	2.08	0	0
C3	07	60	28	30	7.58	2134.92	**30**	31	32	31	31	31			**30**	**30**
	08	60	29	30	8.16	4334.20	**30**	31	34	32	31	32			31	31
	09	60	28	30	8.02	0.05	**30**	**30**	33	31	31	31			**30**	**30**
Average percentage deviation from optimum							**0**	2.15	9.04	4.23	3.23	4.23	3.16	3.16	1.08	1.08
Number of optimal solutions							9/9	5/9	0/9	2/9	3/9	2/9			8/9	8/9

The results for jack and kendall instances from [6] and [56] are showed in Table 2. The last four columns are the solutions obtained by other approaches such as the genetic algorithm combined with a deterministic algorithm proposed by *Jakobs* in [6], a bottom-left algorithm for the genetic algorithm proposed by *Liu and Teng* in [39], the genetic algorithm proposed by *Mumford-Valenzuela* in [63], and the genetic algorithm based on layouts proposed by *Bortfeld* in [41]. These approaches are heuristics, and no execution times were reported. To the best of our knowledge, there are no other exact algorithms that have solved these instances. The P&C is the only method that guarantees the optimal solution (bold numbers) for the three instances, which were solved in less than 70 seconds, considering the generation time of the correspondence matrix and the execution time of the B&B.

Table 3 shows the experimental results for dagli instances. For these instances, we add two columns for the P&C: column “%” represents the packing density which is added to make a comparison with the other approaches, and column “$\bar{LB}$” that shows the best lower bound found by the P&C methodology when an instance is not solved to optimality considering the time limit. The last three columns show the results in terms of the average of packing density for other approaches (no execution times were reported for these algorithms) such as an object-based evolutionary algorithm proposed by *Ratanapan and Dagli* in [54, 55], an artificial neural network, mathematical programming and genetic algorithm proposed by *Dagli and Poshyanonda* in [53] and [64], and a genetic algorithm based on layouts proposed by *Bortfeld* in [41] (we report the best solution obtained by this author). The P&C improves or certifies the optimality of the lower bound in the literature for the four instances. The optimum for dagli01 and dagli03 was not known before of this study according to the Working Group on Cutting and Packing within EURO of the Association of the European Operational Research Societies (ESICUP). The P&C methodology was able to solve 3 of 4 instances to optimality obtaining the best average packings. Furthermore, the P&C improves the LB of the literature for dagli04 instance from 161 to 208. For this instance, our methodology cannot guarantee the optimality because the B&B reaches the time limit with the value of 208. However, it obtains a feasible packing for the value 209 and infeasible for 207. When the lower bound is not close to the optimum, as for dagli04, we searched for the optimal height of the strip in a dichotomous way. Notice that the times of the last iteration of the P&C methodology are less than 15 minutes.

The experimental results for hifi instances proposed by [34] are shown in Table 4. This table is interesting since it compares several dichotomous algorithms based on exact methodologies. The P&C is compared with four approaches of the literature (the last eight columns) such as the dichotomous exact approach based on a B&B and dynamic-programming procedures developed by *Hifi* in [34], the B&B algorithm of [65] called *S*_*bb*_, based on the B&B of [2], the dichotomous algorithm of [65] named *S*_*da*_, based on the one of [34] with the decision version problem solved with the model of [66], and the branch-and-price algorithm of [65] called *S*_*bp*_. For each approach, we present its solution *z* and the execution time ET. Hifi’s algorithm was coded in C and tested on a Sparc-Server20 (module 712 MP). The *S*_*bb*_, *S*_*da*_ and *S*_*bp*_ algorithms were coded in C++ and tested on a Pentium M 1.7 GHz with 1G of RAM. The column generation and linear programs were solved with CPLEX and Concert Technology.

For hifi instances, we are not comparing the dichotomous behavior that seems to be a promising characteristic for the 2SP, but, we are analyzing the exact algorithms that solve the one-dimensional problem. Indeed, the best algorithms in terms of solution value are P&C, *S*_*da*_, and *S*_*bb*_, respectively. To the best of our knowledge, the P&C methodology is the only one finding the optimal solution for instances hifi19 and hifi25. Furthermore, the execution times of the last iteration for the P&C are efficient since they are less than 45 minutes in the worst case (instance hifi18). Nevertheless, the execution times of Hifi’s algorithm seem more competitive.

The experimental results for small-medium size instances proposed by Hopper and Turton in [36] are presented in Table 5. We add a new column at the beginning of the table to specify the class of the instance. We compare the results of the P&C with the hybrid algorithm proposed in [42] (column 9). Columns 10–13 show the results obtained by the best-fit algorithm from [67] and its enhancements adding Tabu-search (TS), simulated-annealing (SA), and a genetic algorithm (GA) from [68]. In columns 14 and 15 are presented the average and best solutions obtained by the genetic algorithm presented in [41], the authors do not present detailed solutions for each instance. Finally, the last two columns show the average and best results obtained by the GRASP proposed by [40]. Numbers in bold represent the optimal solutions. To make a comparison of our methodology with respect to other approaches, we use the average percentage deviation from optimum proposed by [40]. This average is calculated as (sol–opt)/opt. The results show that the P&C algorithm obtains the best solutions for the first three classes (small-medium instances), getting 9 of 9 optimal solutions, improving the best results of the GRASP proposed by [40]. We do not present the results for large instances since P&C cannot obtain their optimal solutions. Indeed, the size of the correspondence matrix for these instances is too large, and the memory of our computer is not enough to solve them.

### Instances for other two-dimensional cutting problems

In this section, we present the experimental results for the other two-dimensional cutting instances which have been adapted to the 2SP (Tables 6–10). The format for each table is similar to the previous ones.

**Table 6:** Results for burke instances.

Inst	Size		Opt	P&C			Best fit	Burke			GRASP		
	*W*	*n*		GT	ET	z*		BF+TS	BF+SA	BF+GA	Average	Best
N1	40	10	40	1.66	3.01	**40**	45	**40**	**40**	**40**	**40**	**40**
N2	30	20	50	2.98	72.64	**50**	53	**50**	**50**	**50**	**50**	**50**
N3	40	30	50	8.31	27.91	**50**	52	51	51	52	51	51
N4	80	40	80	102.85	25047.90	**80**	86	83	82	83	81	81
Number of optimal solutions						4/4	0/4	2/4	2/4	2/4	2/4	2/4

**Table 7:** Results for *ngcut, cgcut*, and *beng* instances.

Class	Inst	Size		LB	P&C			Iori	GRASP	
		*W*	*n*		GT	ET	z*		Average	Best
Beasley *ngcut*	01	10	10	23	0.01	0.03	**23**	**23**	**23**	**23**
	02	10	17	30	0.04	0.14	**30**	**30**	**30**	**30**
	03	10	21	28	0.08	1.02	**28**	**28**	**28**	**28**
	04	10	7	20	0.01	0.01	**20**	**20**	**20**	**20**
	05	10	14	36	0.07	0.37	**36**	**36**	**36**	**36**
	06	10	15	29	0.06	0.42	**31**	**31**	**31**	**31**
	07	20	8	20	0.04	0.00	**20**	**20**	**20**	**20**
	08	20	13	32	0.21	1.04	**33**	**33**	**33**	**33**
	09	20	18	49	0.82	67.47	**50**	**50**	**50**	**50**
	10	30	13	80	1.81	13.98	**80**	**80**	**80**	**80**
	11	30	15	50	1.26	102.08	**52**	**52**	**52**	**52**
	12	30	22	87	3.66	586.74	**87**	**87**	**87**	**87**
Christofides *cgcut*	01	10	16	23	0.04	0.23	**23**	**23**	**23**	**23**
	02	70	23	63	15.09	24314.86	64	65	65	65
	03	70	62	636	MO	TO	–	661	661	
Bengtsson *beng*	01	25	20	30	1.52	40.80	**30**	31	**30**	**30**
	02	25	40	57	11.06	2756.12	**57**	58	**57**	**57**
	03	25	60	84	36.42	8096.72	**84**	86	**84**	**84**
	04	25	80	107	73.53	18104.00	**107**	110	**107**	**107**
	05	25	100	134	130.22	TO	–	136	**134**	**134**
	06	40	40	36	11.12	1110.94	**36**	37	**36**	**36**
	07	40	80	67	75.23	19849.40	**67**	69	**67**	**67**
	08	40	120	101	214.07	TO	–	–	**101**	**101**
	09	40	160	126	379.02	TO	–	–	**126**	**126**
	10	40	200	156	645.63	TO	–	–	**156**	**156**
Number of proven optimal solutions							19/25	13/25	23/25	23/25

**Table 8:** Results for BW instances, class 1.

Inst	Size		LB	P&C					Iori	Bortfeldt		GRASP	
	W	n		$\hat{LB}$	$\bar{LB}$	GT	ET	z*		Average	Best	Average	Best
1	30	20		65	70	0.62	8.45	**70**					
2				44	44	0.30	11.38	**44**					
3				65	73	0.71	17.75	**73**					
4				47	48	0.41	51.00	**48**					
5				54	54	0.48	36.70	**54**					
6				74	77	0.94	19.83	79					
7				53	55	0.47	0.96	**55**					
8				51	52	0.44	7.05	**52**					
9				62	69	0.63	5.93	**69**					
10				67	69	0.71	1.27	**69**					
			60.3	58.2	61.1			61.3	61.2	62.0	61.6	61.3	61.3
11	30	40		90	91	2.15	78.69	**91**					
12				107	108	3.53	98.66	**108**					
13				139	145	6.48	53.89	**145**					
14				125	129	6.07	74.24	**129**					
15				138	140	5.48	336.94	**140**					
16				107	121	4.22	3.37	122					
17				108	109	3.26	41.40	**109**					
18				146	165	7.32	41.05	169					
19				101	102	3.20	80.72	**102**					
20				103	103	3.07	3722.60	**103**					
			121.6	116.4	121.3			121.8	122.1	122.3	122.0	121.9	121.9
21	30	60		201	217	16.94	1140.48	**217**					
22				177	181	11.97	715.23	**181**					
23				182	194	13.39	218.68	**194**					
24				183	209	16.49	3021.05	**209**					
25				165	165	10.32	462.32	**165**					
26				166	168	11.41	488.32	**168**					
27				148	150	10.13	221.44	**150**					
28				189	196	15.84	314.78	**196**					
29				174	175	12.35	745.81	**175**					
30				211	230	18.71	310.80	**230**					
			187.4	179.6	188.5			**188.5**	189.0	189.1	189.0	188.7	188.6
31	30	80		228	240	29.41	90.10	**240**					
32				241	254	28.64	207.28	**254**					
33				230	265	30.90	343.02	**265**					
34				244	261	33.02	1843.17	**261**					
35				236	245	29.65	1316.57	**245**					
36				253	263	32.45	2147.05	**263**					
37				266	289	36.16	2113.05	**289**					
38				265	282	37.09	101.73	**282**					
39				279	282	35.54	26216.00	**282**					
40				243	245	29.50	2707.75	**245**					
			262.2	248.5	262.6			**262.6**	262.8	262.9	262.8	262.9	262.8
41	30	100		274	274	42.60	4015.39	**274**					
42				304	306	47.56	8514.15	**306**					
43				267	267	36.29	5709.47	**267**					
44				289	293	46.68	3592.48	**293**					
45				302	309	49.34	5481.73	**309**					
46				338	355	66.29	3223.22	**355**					
47				274	276	38.73	4500.47	**276**					
48				313	315	57.47	8984.06	**315**					
49				299	300	50.70	7200.16	**300**					
50				342	353	64.95	2556.37	**353**					
			304.4	300.2	304.8			**304.8**	305.5	305.2	305.0	305.6	305.5

**Table 9:** Results for BW instances, class 2.

Inst	Size		LB	P&C					Iori	Bortfeldt		GRASP	
	W	n		$\hat{LB}$	$\bar{LB}$	GT	ET	z*		Average	Best	Average	Best
1	30	20		22	22	0.69	30.60	**22**					
2				15	15	0.34	4.35	**15**					
3				22	22	0.77	45.98	**22**					
4				16	16	0.35	21.28	**16**					
5				18	18	0.46	16.72	**18**					
6				25	25	0.98	23.25	**25**					
7				18	18	0.47	19.43	**18**					
8				17	17	0.45	47.61	**17**					
9				21	21	0.65	17.64	**21**					
10				23	23	0.87	23.34	**23**					
			19.7	19.7	19.7			**19.7**	19.9	20.5	20.5	19.8	19.8
11	30	40		30	30	2.66	62.38	**30**					
12				36	36	4.46	122.07	**36**					
13				47	47	7.10	262.07	**47**					
14				42	42	6.22	200.17	**42**					
15				46	46	6.86	735.38	**46**					
16				36	36	4.14	84.77	**36**					
17				36	36	3.82	99.84	**36**					
18				49	49	7.63	292.17	**49**					
19				34	34	3.73	109.64	**34**					
20				35	35	3.36	84.92	**35**					
			39.1	39.1	39.1			**39.1**	40.0	39.5	**39.1**	**39.1**	**39.1**
21	30	60		67	67	20.07	2311.94	**67**					
22				59	59	16.90	1476.75	**59**					
23				61	61	17.67	1987.65	**61**					
24				61	61	18.67	1985.76	**61**					
25				55	55	12.89	2450.50	**55**					
26				56	56	15.48	1288.01	**56**					
27				50	50	13.39	564.08	**50**					
28				63	63	20.35	2493.82	**63**					
29				58	58	16.27	1784.42	**58**					
30				71	71	27.23	4553.50	**71**					
			60.1	60.1	60.1			**60.1**	61.6	60.5	**60.1**	60.2	**60.1**
31	30	80		76	76	37.64	6942.25	**76**					
32				81	81	40.78	6197.83	**81**					
33				77	77	37.45	6332.58	**77**					
34				82	82	42.84	6223.78	**82**					
35				79	79	37.46	4777.47	**79**					
36				85	85	39.72	12458.30	**85**					
37				89	89	45.45	17841.10	**89**					
38				89	89	49.60	7866.01	**89**					
39				93	93	58.32	14936.60	**93**					
40				81	81	43.05	7035.82	**81**					
			83.2	83.2	83.2			**83.2**	84.7	83.4	83.3	**83.2**	**83.2**
41	30	100		92	92	63.05	8929.45	**92**					
42				102	102	70.02	10779.90	**102**					
43				89	89	52.28	11173.80	**89**					
44				97	97	68.12	18199.50	**97**					
45				101	101	74.76	15758.60	**101**					
46				113	113	93.92	22739.10	**113**					
47				92	92	59.66	10663.10	**92**					
48				105	105	84.50	16105.70	**105**					
49				100	100	78.56	15360.00	**100**					
50				114	114	97.13	23182.70	**114**					
			100.5	100.5	100.5			**100.5**	101.8	100.7	100.7	**100.5**	**100.5**

**Table 10:** Results for kenmochi instances.

Inst	Size		P&C				Kenmochi		
	*W*	*n*	*W*	GT	ET	z*	BL+DP	BL+DP+LP	S+DP
1	13	9	**15**	0.08	0.14	**20**	20	20	20
2	13	9	**15**	0.08	0.00	**20**	20	20	20
3	20	10	20	0.25	0.19	**23**	20	20	20
4	20	11	20	0.26	1.13	**22**	20	20	20
5	20	12	20	0.27	1.37	**21**	20	20	20
6	20	13	20	0.25	0.00	**22**	20	20	20
Number of optimal solutions						6/6			

In Table 6, we show the results for the burke instances of [67]. We compare the P&C with the best-fit algorithm from [67] and its enhancements adding Tabu-search (TS), simulated-annealing (SA), and a genetic-algorithm (GA) from [68] (columns 8–11). Finally, the last two columns show the average and best results obtained for the GRASP proposed by [40]. Notice that the P&C is the only approach that obtains the optimal solutions for all the instances.

Table 7 shows the experimental results for *ngcut*, *cgcut*, and *beng* instances proposed by [58, 59], and [61], respectively. For these instances, the P&C methodology is compared with the algorithms of [42] and the GRASP of [40]. MO means that the computer memory was not enough to generate the correspondence matrix, and TO means the time limit to solve the instances was reached. The best results were obtained for the GRASP algorithm of [40]. However, our contribution here is that we have increased and validated the optimal solutions reported by the GRASP from 19/25 to 23/25. For the *cgcut_02* instance, the P&C cannot prove its optimal solution since the model reached the time-limit when the lower bound was used as the maximum height of the strip. However, the P&C obtains the best result for this instance with respect to the other approaches.

Tables 8 and 9 present the experimental results for Class 1 and 2 of Berkey and Wang instances proposed in [57]. For each table, we add two new columns for the P&C. Column $\hat{LB}$ represents the initial lower bound computed with Eq (1) and used to start our algorithm. Column $\bar{LB}$ shows the best lower bound found by the P&C when an instance is not solved to optimality (considering the time limit established for the B&B).

As we mention in the description of the instances, each class has five groups of ten instances that differ one with each other in the number of items. Therefore, both tables are divided into five sections that represent the corresponding group of instances for each class. For each group, we show the average to compare our results with other approaches of the literature. For Class 1, the P&C cannot solve to optimality instances 6, 16, and 18. However, our methodology can find new lower bounds for the first two groups of instances, improving the bounds given in the literature. For the last three groups of Class 1, the P&C can solve all the instances to optimality, improving the lower bounds and the results of other methodologies. Finally, for Class 2, the P&C solved all the instances to optimality, improving the results of the GRASP.

Table 10 shows the results for kenmochi instances proposed by [33]. The first three columns describe the instance as usual. Columns 4–7 show the results obtained with the P&C. In this case, we show the width *W* (the fourth column) and the optimal height z* of the strip because some anomalies exist in the instances, and a perfect packing does not exist with the original width. The anomalies exist in the first two instances where the width of some items is larger than the width of the bin. Therefore, to solve these instances, we set the width of the bin as long as the width of the larger item (numbers in bold in column 4 show the modification of *W*). Furthermore, the optimal solutions obtained by our methodology in the last four instances do not correspond with the results presented by Kenmochi [33]. Columns 8–10 present the results of [33] that implement two branching strategies: the *bottom-left (BL)* point and the the *staircase (S)* strategy. The bounding operations are based on *dynamic programming (DP)* and *linear programming (LP)*. The P&C shows the optimal results for all the instances.

The experimental results show that the P&C is an efficient methodology to solve small-medium size instances for the 2SP. Larger instances cannot be solved with the P&C methodology since the combinatorial complexity of the problem also relies on the generation of all the possible positions of the items. In this case, we have detected an opportunity area where we propose to apply a decomposition approach, such as a column generation, to obtain optimal solutions for large instances.

## Conclusion

In this study, we present a new methodology called “Positions and Covering (P&C)” to obtain exact solutions for the two-dimensional strip packing problem. The methodology is based on a two-stage procedure where first, a set of valid positions is generated in a pseudo-polynomial way representing how each item can be allocated inside of the strip. The height of the strip is computed assuming that a perfect packing exists (or using the best lower bound, if it exists). In the second stage, a set covering formulation (using the set of valid positions) is solved to determine if the height of the strip is optimal. If the mathematical model is feasible, then the procedure ends with the optimal solution. Else, the height of the strip is increased by one, a new set of valid positions is generated, and a new iteration begins.

The P&C methodology was tested using the benchmark for the 2SP solving small and medium-size instances. We verify that many of the solutions proposed by other approaches were the optimal solutions. The main contribution of this study is that we have obtained optimal solutions for instances proposed in the literature where the optimum was not known before. Furthermore, we have modified some other instances of the literature, which were infeasible, and we have solved them, obtaining the optimal solutions.

An essential characteristic of the P&C is its simple implementation. Nevertheless, a future research line consists of developing a decomposition approach to enhance the P&C methodology to give optimal solutions for larger instances of the 2SP.

## References

[1] Lesh N, Marks J, McMahon A, Mitzenmacher M. Exhaustive approaches to 2D rectangular perfect packings. Information Processing Letters. 2004 4;90(1):7–14. 10.1016/j.ipl.2004.01.006

[2] Martello S, Monaci M, Vigo D. An exact approach to the strip-packing problem. INFORMS Journal on Computing. 2003 8;15(3):310–319. 10.1287/ijoc.15.3.310.16082

[3] Baker BS, Coffman EG Jr, Rivest RL. Orthogonal packings in two dimensions. SIAM Journal on Computing. 1980 3;9(4):846–855. 10.1137/0209064

[4] Hochbaum DS, Maass W. Approximation schemes for covering and packing problems in image processing and VLSI. Journal of the ACM (JACM). 1985 1;32(1):130–136. 10.1145/2455.214106

[5] Wäscher G, Haußner H, Schumann H. An improved typology of cutting and packing problems. European Journal of Operational Research. 2007 12;183(3):1109–1130. 10.1016/j.ejor.2005.12.047

[6] Jakobs S. On genetic algorithms for the packing of polygons. European Journal of Operational Research. 1996 1;88(1):165–181. 10.1016/0377-2217(94)00166-9

[7] Gilmore P, Gomory RE. Multistage cutting stock problems of two and more dimensions. Operations Research. 1965 2;13(1):94–120.

[8] Hopper E, Turton B. A review of the application of meta-heuristic algorithms to 2D strip packing problems. Artificial Intelligence Review. 2001 12;16(4):257–300. 10.1023/A:1012590107280

[9] Augustine J, Banerjee S, Irani S. Strip packing with precedence constraints and strip packing with release times. In: Proceedings of the eighteenth annual ACM symposium on Parallelism in algorithms and architectures. ACM; 2006. p. 180–189.

[10] Cid-Garcia NM, Rios-Solis YA Positions and covering: A two-stage methodology to obtain optimal solutions for the 2d-bin packing problem. Plos one. 2020 4;15(4):e0229358 10.1371/journal.pone.0229358 32251428 PMC7135247

[11] Albornoz VM, Cid-Garcia NM, Ortega R, Rios-Solis YA. A Hierarchical Planning Scheme Based on Precision Agriculture In: Plà -Aragonés L, editor. The Handbook of Operations Research in Agriculture and the Agri-Food Industry. New York: Springer; 2015 p. 129–162.

[12] Cid-Garcia NM, Albornoz V, Rios-Solis YA, Ortega R. Rectangular shape management zone delineation using integer linear programming. Computers and Electronics in Agriculture. 2013 4;93:1–9. 10.1016/j.compag.2013.01.009

[13] Cid-Garcia NM, Ibarra-Rojas OJ. An integrated approach for the rectangular delineation of management zones and the crop planning problems. Computers and Electronics in Agriculture. 2019 9;164:104925 10.1016/j.compag.2019.104925

[14] Bayliss C, Currie CSM, Bennell JA, Martinez-Sykora A. Queue-constrained packing: A vehicle ferry case study. European Journal of Operational Research. 2021 3;289(2):727–741. 10.1016/j.ejor.2020.07.027

[15] Fırat H, Alpaslan N. An effective approach to the two-dimensional rectangular packing problem in the manufacturing industry. Computers & Industrial Engineering. 2020 10;148:106687 10.1016/j.cie.2020.106687

[16] Tapia JFD, Lee J, Ooi REH, Foo DCY, Tan RR. Planning and scheduling of CO2 capture, utilization and storage (CCUS) operations as a strip packing problem. Process Safety and Environmental Protection. 2016 11;104(A):358–372. 10.1016/j.psep.2016.09.013

[17] Abedini A, Ye H, Li W. Operating room planning under surgery type and priority constraints. Procedia Manufacturing. 2016; 5:15–25. 10.1016/j.promfg.2016.08.005

[18] Li F, Gupta D, Potthoff S. Improving operating room schedules. Health care management science. 2016 2;19(3):261–278. 10.1007/s10729-015-9318-2 25687390

[19] Vijayakumar B, Parikh PJ, Scott R, Barnes A, Gallimore J. A dual bin-packing approach to scheduling surgical cases at a publicly-funded hospital. European Journal of Operational Research. 2013 2;224(3):583–591. 10.1016/j.ejor.2012.09.010

[20] Aydin N, Muter I, Birbil SI. Multi-objective temporal bin packing problem: An application in cloud computing. Computers & Operations Research. 2020 9;121:104959 10.1016/j.cor.2020.104959

[21] Feng D, Wu Z, Zuo D, Zhang Z. A multiobjective migration algorithm as a resource consolidation strategy in cloud computing. PloS one. 2010 2;14(2):e0211729 10.1371/journal.pone.0211729 PMC6364968 30726283

[22] Ye D, Xie F, Zhang G. Truthful mechanism design for bin packing with applications on cloud computing. Journal of Combinatorial Optimization. 2020 10.1007/s10878-020-00601-4

[23] Rhiat A, Aggoun A, Lachere R. Combining Mobile Robotics and Packing for Optimal deliveries. Procedia Manufacturing. 2020;44:536–542. 10.1016/j.promfg.2020.02.258

[24] Ntene N, van Vuuren JH. A survey and comparison of guillotine heuristics for the 2d oriented offline strip packing problem. Discrete Optimization. 2009 5;6(2):174–188. 10.1016/j.disopt.2008.11.002

[25] Riff MC, Bonnaire X, Neveu B. A revision of recent approaches for two-dimensional strip-packing problems. Engineering Applications of Artificial Intelligence. 2009 6;22(4-5):823–827. 10.1016/j.engappai.2008.10.025

[26] Dowsland KA, Dowsland WB. Packing problems. European Journal of Operational Research. 1992 1;56(1):2–14. 10.1016/0377-2217(92)90288-K

[27] Haessler RW, Sweeney PE. Cutting stock problems and solution procedures. European Journal of Operational Research. 1991 9;54(2):141–150. 10.1016/0377-2217(91)90293-5

[28] Lodi A, Martello S, Monaci M. Two-dimensional packing problems: A survey. European Journal of Operational Research. 2002 9;141(2):241–252. 10.1016/S0377-2217(02)00134-0

[29] Lodi A, Martello S, Vigo D. Recent advances on two-dimensional bin packing problems. Discrete Applied Mathematics. 2002 11;123(1–3):379–396. 10.1016/S0166-218X(01)00347-X

[30] Alvarez-Valdes R, Parreno F, Tamarit J. A branch and bound algorithm for the strip packing problem. OR spectrum. 2009 4;31(2):431–459. 10.1007/s00291-008-0128-5

[31] Arahori Y, Imamichi T, Nagamochi H. An exact strip packing algorithm based on canonical forms. Computers & Operations Research. 2012 12;39(12):2991–3011. 10.1016/j.cor.2012.03.003

[32] Boschetti MA, Montaletti L. An exact algorithm for the two-dimensional strip-packing problem. Operations Research. 2010 12;58(6):1774–1791. 10.1287/opre.1100.0833

[33] Kenmochi M, Imamichi T, Nonobe K, Yagiura M, Nagamochi H. Exact algorithms for the two-dimensional strip packing problem with and without rotations. European Journal of Operational Research. 2009 10;198(1):73–83. 10.1016/j.ejor.2008.08.020

[34] Hifi M. Exact algorithms for the guillotine strip cutting/packing problem. Computers & Operations Research. 1998 11;25(11):925–940. 10.1016/S0305-0548(98)00008-2

[35] Chazelle B. The bottomn-left bin-packing heuristic: An efficient implementation. IEEE Transactions on Computers. 1983 8;C-32(8):697–707. 10.1109/TC.1983.1676307

[36] Hopper E, Turton B. 2001. An empirical investigation of meta-heuristic and heuristic algorithms for a 2d packing problem. European Journal of Operational Research. 2001 1;128(1):34–57. 10.1016/S0377-2217(99)00357-4

[37] Gonçalves JF, Resende MG. A biased random key genetic algorithm for 2D and 3D bin packing problems. International Journal of Production Economics. 2013 10;145(2):500–510. 10.1016/j.ijpe.2013.04.019

[38] Lesh N, Marks J, McMahon A, Mitzenmacher M. New heuristic and interactive approaches to 2d rectangular strip packing. Journal of Experimental Algorithmics (JEA). 2005 12;10:1–2.

[39] Liu D, Teng H. An improved BL-algorithm for genetic algorithm of the orthogonal packing of rectangles. European Journal of Operational Research. 1999 1;112(2):413–420. 10.1016/S0377-2217(97)00437-2

[40] Alvarez-Valdés R, Parreño F, Tamarit JM. Reactive grasp for the strip-packing problem. Computers & Operations Research. 2008 4;35(4):1065–1083. 10.1016/j.cor.2006.07.004

[41] Bortfeldt A. A genetic algorithm for the two-dimensional strip packing problem with rectangular pieces. European Journal of Operational Research. 2006 8;172(3):814–837. 10.1016/j.ejor.2004.11.016

[42] Iori M, Martello S, Monaci M. Metaheuristic algorithms for the strip packing problem In: Pardalos PM, Korotkikh V, editors. Optimization and Industry: New frontiers. Applied Optimization. New York: Springer; 2003 p. 159–179.

[43] Lodi A, Martello S, Vigo D. TSpack: a unified tabu search code for multi-dimensional bin packing problems. Annals of Operations Research. 2004 10;131(1–4):203–213. 10.1023/B:ANOR.0000039519.03572.08

[44] Rakotonirainy RG, van Vuuren JH. Improved metaheuristics for the two-dimensional strip packing problem. Applied Soft Computing. 2020 7; 92:106268 10.1016/j.asoc.2020.106268

[45] Bezerra VMR, Leao AAS, Oliveira JF, Santos MO. Models for the two-dimensional level strip packing problem–a review and a computational evaluation. Journal of the Operational Research Society. 2020 4;71(4):606–627. 10.1080/01605682.2019.1578914

[46] Wei L, Hu Q, Leung SCH, Zhang N. An improved skyline based heuristic for the 2D strip packing problem and its efficient implementation. Computers & Operations Research. 2017 4; 80:113–127. 10.1016/j.cor.2016.11.024

[47] Júnior AN, Silva E, Gomes AM, Soares C, Oliveira JF. Data mining based framework to assess solution quality for the rectangular 2D strip-packing problem. Expert Systems with Applications. 2019 3; 118:365–380. 10.1016/j.eswa.2018.10.006

[48] Chen M, Li K, Zhang D, Zheng L, Fu X. Hierarchical Search-Embedded Hybrid Heuristic Algorithm for Two-Dimensional Strip Packing Problem. IEEE Access. 2019 11; 7:179086–179103. 10.1109/ACCESS.2019.2953531

[49] Maschler J, Raidl GR. A logic-based Benders decomposition approach for the 3-staged strip packing problem In: Dörner K, Ljubic I, Pflug G, Tragler G, editors. Operations Research Proceedings 2015. Springer; 2017 p. 393–399.

[50] Sugi M, Shiomi Y, Okubo T, Nagai H, Inoue K, Ota J. Solution of the Rectangular Strip Packing Problem Considering a 3-Stage Guillotine Cutting Constraint with Finite Slitter Blades. International Journal of Automation Technology. 2020 5; 14(3):447–458. 10.20965/ijat.2020.p0447

[51] Oliveira JF, Neuenfeldt Júnior A, Silva E, Carravilla MA. A survey on heuristics for the two-dimensional rectangular strip packing problem. Pesquisa Operacional. 2016 May-Aug; 36(2):197–226. 10.1590/0101-7438.2016.036.02.0197

[52] Buchwald T, Scheithauer G. Upper bounds for heuristic approaches to the strip packing problem. International Transactions in Operational Research. 2016 5; 23(1-2):93–119. 10.1111/itor.12100

[53] Dagli CH, Poshyanonda P. New approaches to nesting rectangular patterns. Journal of Intelligent Manufacturing. 1997 5;8(3):177–190. 10.1023/A:1018517106992

[54] Ratanapan K, Dagli C. An object-based evolutionary algorithm for solving irregular nesting problems. In: Proceedings for Artificial Neural Networks in Engineering Conference. ANNIE’97; 1997. p. 383–388.

[55] Ratanapan K, Dagli C. An object-based evolutionary algorithm: the nesting solution. In: 1998 IEEE International Conference on Evolutionary Computation Proceedings. IEEE World Congress on Computational Intelligence (Cat. No. 98TH8360); 1998. p. 581–586.

[56] Burke E, Kendall G. Applying simulated annealing and the no fit polygon to the nesting problem In: Proceedings of the world manufacturing congress. Citeseer; 1999 p. 27–30.

[57] Berkey JO, Wang PY. Two-dimensional finite bin-packing algorithms. Journal of the Operational Research Society. 1987 5;38(5):423–429. 10.1057/jors.1987.70

[58] Christofides N, Whitlock C. An algorithm for two-dimensional cutting problems. Operations Research. 1977 2;25(1):30–44. 10.1287/opre.25.1.30

[59] Beasley JE. An exact two-dimensional non-guillotine cutting tree search procedure. Operations Research. 1985 2;33(1):49–64. 10.1287/opre.33.1.49

[60] Martello S, Vigo D. Exact solution of the two-dimensional finite bin packing problem. Management Science. 1998 3;44(3):388–399. 10.1287/mnsc.44.3.388

[61] Bengtsson BE. Packing rectangular pieces—A heuristic approach. The computer journal. 1982 8;25(3):353–357. 10.1093/comjnl/25.3.353

[62] Fekete SP, Schepers J, Van der Veen JC. An exact algorithm for higher-dimensional orthogonal packing. Operations Research. 2007 6;55(3):569–587. 10.1287/opre.1060.0369

[63] Mumford-Valenzuela CL, Vick J, Wang PY. Heuristics for large strip packing problems with guillotine patterns: An empirical study In: Resende MG, de Sousa JP, editors. Metaheuristics: computer decision-making. Applied Optimization, vol 86 Boston, MA: Springer; 2003 p. 501–522.

[64] Poshyanonda P, Dagli C. Genetic neuro-nester. Journal of Intelligent Manufacturing. 2004 4; 15(2):201–218. 10.1023/B:JIMS.0000018033.05556.65

[65] Bekrar A, Kacem I, Chu C. A comparative study of exact algorithms for the two dimensional strip packing problem. Journal of Industrial and Systems Engineering. 2007;1(2):151–170.

[66] Pisinger D, Sigurd M. The two-dimensional bin packing problem with variable bin sizes and costs. Discrete Optimization. 2005 6; 2(2):154–167. 10.1016/j.disopt.2005.01.002

[67] Burke EK, Kendall G, Whitwell G. A new placement heuristic for the orthogonal stock-cutting problem. Operations Research. 2004 8;52(4):655–671.

[68] Burke EK, Kendall G, Whitwell G. Metaheuristic enhancements of the best-fit heuristic for the orthogonal stock cutting problem. Computer Science Technical Report No. NOTTCS-TR-2006-3. University of Nottingham; 2006.

